# Spatial Structure of Above-Ground Biomass Limits Accuracy of Carbon Mapping in Rainforest but Large Scale Forest Inventories Can Help to Overcome

**DOI:** 10.1371/journal.pone.0138456

**Published:** 2015-09-24

**Authors:** Stéphane Guitet, Bruno Hérault, Quentin Molto, Olivier Brunaux, Pierre Couteron

**Affiliations:** 1 Office National des Forêts (ONF), R&D department, Cayenne, French Guiana; 2 Institut National de la Recherche Agronomique (INRA), UMR Amap, Montpellier, France; 3 Centre de coopération Internationale de la Recherche Agronomique pour le Développement (CIRAD), UMR EcoFoG, Kourou, French Guiana; 4 CIRAD, UR BSEF, Montpellier, France; 5 Institut de Recherche pour le Développement (IRD), UMR Amap, Montpellier, France; University of Maryland at College Park, UNITED STATES

## Abstract

Precise mapping of above-ground biomass (AGB) is a major challenge for the success of REDD+ processes in tropical rainforest. The usual mapping methods are based on two hypotheses: a large and long-ranged spatial autocorrelation and a strong environment influence at the regional scale. However, there are no studies of the spatial structure of AGB at the landscapes scale to support these assumptions. We studied spatial variation in AGB at various scales using two large forest inventories conducted in French Guiana. The dataset comprised 2507 plots (0.4 to 0.5 ha) of undisturbed rainforest distributed over the whole region. After checking the uncertainties of estimates obtained from these data, we used half of the dataset to develop explicit predictive models including spatial and environmental effects and tested the accuracy of the resulting maps according to their resolution using the rest of the data. Forest inventories provided accurate AGB estimates at the plot scale, for a mean of 325 Mg.ha^-1^. They revealed high local variability combined with a weak autocorrelation up to distances of no more than10 km. Environmental variables accounted for a minor part of spatial variation. Accuracy of the best model including spatial effects was 90 Mg.ha^-1^ at plot scale but coarse graining up to 2-km resolution allowed mapping AGB with accuracy lower than 50 Mg.ha^-1^. Whatever the resolution, no agreement was found with available pan-tropical reference maps at all resolutions. We concluded that the combined weak autocorrelation and weak environmental effect limit AGB maps accuracy in rainforest, and that a trade-off has to be found between spatial resolution and effective accuracy until adequate “wall-to-wall” remote sensing signals provide reliable AGB predictions. Waiting for this, using large forest inventories with low sampling rate (<0.5%) may be an efficient way to increase the global coverage of AGB maps with acceptable accuracy at kilometric resolution.

## Introduction

Estimating carbon flux due to afforestation, deforestation, and forest degradation requires quantifying above-ground biomass (AGB), especially over extensive areas of old-growth tropical forests which have high but varied carbon stocks and are threatened by a rapidly changing land-use dynamics in many countries [1]. Precise mapping of AGB in tropical rainforest is a thus major challenge for the success of REDD+ processes [2]. The objectives set by international organizations are very ambitious but they are faced the inability of many tropical countries to produce accurate maps of AGB [3]. In fact, in most countries where land-use changes and forestry are major contributors to greenhouse emissions, biomass pools are poorly reported (i.e. use the *tier 1* default value proposed by IPCC—Intergovernmental Panel on Climate Change), whereas precise estimates based on specific spatial data are required (i.e. IPCC tier 2 and tier 3 methods [2]).

Many mapping methods based on forest inventories and/or remote-sensing products have been developed in recent decades [4]. The main techniques, whose applications are not mutually exclusive and are sometimes combined, are based on: (i) spatial interpolation between forest plots, generally by inverse-distance weighting [5–7]; (ii) deterministic models using stratification (vegetation maps) or previously mapped predictive ecological variables which are assumed to influence forest structure and composition [8]; (iii) remote-sensing approaches, which make it possible to define more homogeneous forest types and/or more efficiently describe the spatial variation of ecological variables [9,10]. These methods imply heavy hypotheses in terms of biomass distribution, which need to be corroborated *a posteriori*. Spatial interpolation implies that biomass has a strong spatial structure (i.e. is strongly auto-correlated), and deterministic modelling implies that biomass is influenced to varying degrees by the environment. Remote-sensing approaches often rely on a different base, and aim at more or less direct measurement. The most recent methodological improvements involve remote-sensing data at very high spatial resolution (VHRS), especially LiDAR, which are able to provide direct descriptors of forest structure including tree height, crown size, and tree density, i.e. the main parameters needed to predict biomass [11,12]. Stand level models analogous to forestry allometries are then calibrated to directly convert the physical signals into biomass or carbon estimates [13,14]. However, the coverage of these VHRS images is usually limited by small swaths and their high cost per hectare covered. Consequently, they are frequently combined with medium to coarse resolution data which make it possible to upscale local estimates (based on field data, VHR remote sensing or both) over broader areas [15,16] through the same a *priori* hypotheses (i.e. autocorrelation and dependence on the environment). Several products have already been developed at continental scales using these latter methods. They have been described as a robust basis for national carbon inventories or regional REDD+ projects [15,16].

In spite of this progress, the reliability of most of these mapping products has been shown to be questionable. The precision reported for recent maps varied dramatically between 25 and 65 tC.ha^-1^ (i.e. 50 to 130 Mg.ha^-1^ for AGB) depending on the resolution of the output map, the extent of the area, and the type of vegetation cover (see Table 1), but comparisons with independent validation data often revealed larger bias than the originally reported accuracy [7]. Several problems that limit the reliability of the maps have already been identified: (i) saturation phenomena with certain RS captors at more than 150 t. ha^-1^ [17] (ii) spatial mismatches between field data (located with GPS) and geo-referenced RS images [18]; (iii) problem of representativeness of the calibration data due to small plots, usually < 0.25 ha [19,20]; (iv) « dilution bias » when up-scaling from plot areas to VHRS image footprints, due to local heterogeneity and rugged relief [21]; (v) huge uncertainties at the landscape scale linked to poor interpolation of scarce field data [22]. Most of these pitfalls and biases are linked to the autocorrelation hypothesis and to the lack of representativeness of field data, which are generally undersized, mismatched, and above all too scarce and scattered, given the high variability of forest structure at both local and landscape scales [22]. The spatial structure of biomass at varying nested scales is key information when designing efficient sampling to ensure the robustness of calibration. This issue has been widely studied locally [21,23], but studies at the landscapes scale are lacking [22] because they require the collection of numerous data, which is costly.

**Table 1:** Overview of recent articles focused on “mapping biomass in tropical forest”.

Reference ^a^	Context				Data used for AGB measurement		Predictive variables used for modelling			Model		
	Locality	Cover (ha)	Main vegetation types ^b^	Resolution	Field plot (ha)	Very High Remote Sensing	Remote sensing data	GIS layers	space	Allometry	Predicted range	RMSE (Mg.ha^-1^)
[8]	Rondônia (Brazil)	2.4 M	old forest	1 km	330 x1 ha	no	SRTM	Habitats, soils	no	f(DBH)	100–600	49
									yes	f(DBH)	100–600	35
[27]	Africa	20 M	various	1 km	various	no	MODIS	no	no	f(DBH)	0–350	50.5
[28]	Costa Rica	800	various	30 m	83 x 0.09 ha	LiDAR	no	no	yes	f(DBH)	0–500	38
[12]	Panama	50	old forest	30 m	128 x 0.36 ha	LiDAR	no	no	no	f(DBH,H,WD)	0–400	34
		1,256	various	30 m	128 x 0.36 ha	LiDAR	no	no	no	f(DBH,H,WD)	100–400	38
[29]	Cameroon	1.5 M	various	100 m	8x1 + 10x0.4 ha	PALSAR+JERS	no	no	no	f(DBH,H,WD)	0–400	49
[16] in [7]	Amazonia	423 M	old forest	1 km	493x(≤1ha)	GLASS	MODIS	no	no	varied	50–350	77
	Guiana shield	32 M	old forest	1 km	493x(≤1ha)	GLASS	MODIS	no	no	varied	50–350	123
[15] in [7]	Amazonia	423 M	old forest	500 m	283x0.36 ha	GLASS	MODIS, SRTM, QSCAT	no	no	f(DBH,H,WD)	50–350	83
	Guiana shield	32 M	old forest	500 m	283x0.36 ha	GLASS	MODIS, SRTM, QSCAT	no	no	f(DBH,H,WD)	50–350	117
[30]	Colombia	16.5 M	old forest	100 m	11x0.28 ha	LiDAR	LANDSAT, SRTM	no	no	f(DBH,H,WD)	0–280	56
				30 m	11x0.28 ha	LiDAR	LANDSAT, SRTM	no	no	f(DBH,H,WD)	0–280	82
[31]	Ghats (India)	3 k	old forest	125 m	15x1 ha	no	Google Earth	no	no	f(DBH)	50–650	80
							Ikonos	no	no	f(DBH)	50–650	77
[32]	E. Kalimantan (Indonesia)	83 k	old forest	30 m	77x0.05 ha	no	LANDSAT	no	no	f(DBH)	100–600	130
[33]	Indonesia	10 M	various	200 m	85x0.25 ha	no	MODIS, LANDSAT	no	no	f(DBH,H,WD)	0–450	85
[34]	Borneo	28 k	old forest	20–30 m	48x0.09 ha	LiDAR	no	no	no	f(DBH,H)	50–600	61
[35]	Western Amazon	16 M	various	100 m	214x(≤1ha)	LiDAR	LANDSAT, SRTM, MODIS, TRMM	Habitats, geology	no	f(TCH)	0–300	66
									yes	f(TCH)	0–300	53

^a^ only articles which provided precise information simultaneously on root mean square error (RMSE), resolution, and extent are included.

^b^ “various vegetation types” means the study included explicit samples in savannahs, young plantations or opened/highly degraded forest in addition to forests; “old forest” means the studies focused mainly on old-growth forest (and did not include samples of other vegetation types for calibration and validation).

^c^ DBH for diameter at breast height, H for Height, WD for Wood density or Wood Specific Gravity, TCH for “top-of-canopy height”

Following the development of forestry in tropical countries, more and more forest management inventories are being produced by public and private operators and cover large areas, especially when results from concession-scale operations are lumped together (e.g. [24–26]). However, the measurements are often not sufficiently precise (local vernacular names are used instead of botanical names, diameter at breast height is recorded by class, and there is no measurement of total height). Consequently the uncertainty due to inaccuracy needs to be more precisely assessed but the high repetition rate of the data could compensate for the lack of precision and provide information on the spatial structure of the biomass at landscape scale, as well as solve the problem of representativeness.

For the present study, we used two large forest inventories conducted in French Guiana during the periods 1974–1976 and 2006–2012 in 2,507 plots that sampled 1,120 ha over the 8 M ha undisturbed rainforest. Our aims were to (i) assess the precision of biomass estimates obtained from this kind of forest inventory data; (ii) test the spatial structure of biomass (i.e. auto-correlation in biomass distribution) at various scales in order to assess accuracy as a function of resolution; (iii) produce maps at different resolutions using different predictive models and compare their accuracy with other products, for practical use in REDD+ programmes.

## Materials and Methods

### Field measurements

**Fig 1:**
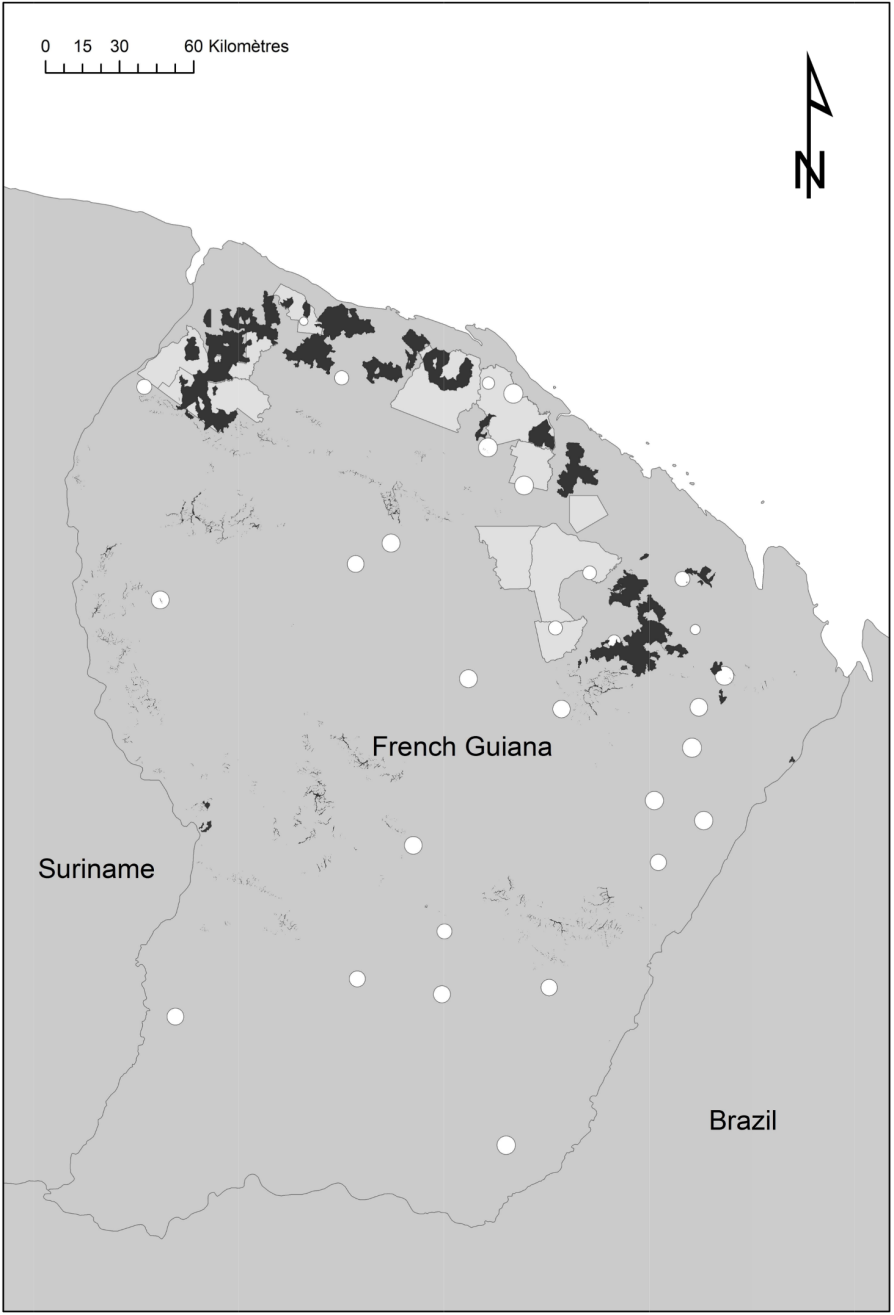
Spatial distribution of inventory blocks from CTFT (1974–1976) and ONF (2006–2013). Inventory blocks from CTFT (1974–1976) in pale grey polygons. Complementary inventory campaigns from ONF (2006–2013) in white circles (size represent the effective area covered by transects). Areas disturbed by harvesting or mining between 1974 and 2007 (in black) were removed from the dataset.

We used two different forest inventories produced by French public organizations **(Fig 1)**. The first inventory was done by CTFT (*Centre Technique Forestier Tropical*) between 1974 and 1976 in the northern part of the French Guiana [36]. CTFT data were scanned between 2006 and 2010 and positioned on GIS using original maps. This dataset corresponded to 126,880 trees (DBH≥20cm) in 1,172 plots 0.5 ha in size.

The second inventory was done between 2006 and 2013 on behalf of ONF (“*Office National des Forêts*”: the French national forest agency) to complete regional coverage and better sample environmental variability [37]. Thirty three sites were selected mostly in the south and east of French Guiana to cover the geological and climatic conditions poorly sampled by the former CTFT inventories. ONF data represented a total of 1,335 0.4-ha plots where 83,075 trees (DBH≥20cm) were measured. All the plots were geo-referenced using a GPS receiver.

These inventories did not include palms and small trees (DBH<20cm). However, they previously proved to represent minor and reasonably stable proportions of total AGB from 10 to 14% (e.g. [38,39]). Details about these two inventories and the pre-treatments used to control their quality and homogenise the two datasets are given in S1 Text.

### Environmental data

For all plots, we extracted from GIS all environmental variables assumed to influence forest growth that were freely accessible on available maps (Table 2). For continuous variables, we computed the mean values over the plot area, while for categorical variables we selected the majority class. We used a simplified geological map [40] for the substrate; SRTM from NASA [41] to produce topographical indices including altitude (ALT), slope (SLO), area of the hydraulic basin in log-scale (LOG) and height above the nearest drainage (i.e. HAND [42]); recent geomorphological maps generated from SRTM [43] to describe landforms and landscapes; a broad-scale vegetation map based on SPOT-VEGETATION remote sensing images [44]; dry season length (DRY) and annual rainfall (RAIN) from TRMM data resampled at 90 m with a bi-cubic method for climatic descriptors [45].

**Table 2:** Environmental variables tested to predict aboveground biomass.

Theme	Description of variables for selected plots	Source	Resolution
Topography	**SLO**pe: 0–142%	[41]	<100 m
	**ALT**itude: 1–819 m	[41]	<100 m
Hydrography	**LOG**arithm of basin area: 0–6.79	[41]	<100 m
	**H**eight **A**bove **N**earest **D**rainage: 0–214 m	[41]; [42]	<100 m
Climate	Annual **RAIN**fall: 2197–3211 mm/y	[45]	<100 m
	**DRY** season length: 1–3 months	[45]	<100 m
Vegetation	**5 VEGET**ation classes - V18: Low dense forest [13%]; V19: High forest with regular canopy [65%]; 20: High forest with disrupted canopy [6%]; 21: Mixed high and open forest [16%]; 22: Open/palm forest [<1%]	[44]	1 km² (Raster)
Geomorphology	**10 LANDF**orms categories – 1: large flat relief [23%]; 3: small flat relief [1%]; 4: small rounded hill [12%]; 5: small flattened hill [2%]; 6: low half-orange [2%]; 7: large high hill [10%]; 8: mountain [8%]; 9: typical half-orange [14%]; 11: wetland [1%]; 12: hillock in lowland[9%]; 13: hillock in highland [3%]; 14: large flat relief [2%]; 15: large rounded hill [13%]	[43]	<10 km² (Vector)
	**10 LANDS**capes categories**–**A: plains [13%], B: irregular multiconvex [13%]; C: valleys [18%]; D: multiconcave [4%]; E: regular plateau [9%]; F: irregular plateau [2%]; G: dissected plateau [7%]; H: mountainous [16%]; I: moderate multiconvex [4%]; J: irregular multiconvex [14%]	[43]	>10 km² (Vector)
Geology	**8 GEOL**ogical substrates**—**G1: Recent sediments [9%]; G2: Dikes [2%]; G3: Various granites [23%]; G4: granodiorites gneiss [21%; G5: Gabbros [10%]; G6: sandstone [2%]; G7: Volcanic sedimentary rock [25%]; G8: Pelites [8%]	[46]	>10 km² (Vector)

### Biomass estimates and uncertainties

We computed above-ground tree biomass (AGB_tree_) using the generic pan-tropical allometry (Eq 1) from Chave et al. [47]. As the forest inventories provided approximate data (i.e. class of DBH instead of precise DBH, no measure for individual height, and vernacular names instead of precise botanical determination to predict wood density), we estimated the uncertainties due to measurement errors. For each tree in a given DBH class, we simulated precise DBH values (D_DBHclass_) using exponential distribution. Next we sampled height value (H_DBHclass/plot_) using a local allometry based on an asymptotic model [48] and calibrated with the plot’s stand structure [48] (see S2 Text). We also allocate trees to precise botanical species using a Monte-Carlo process based on empirical relationships between vernacular names and botanical species, which also accounts for the expected precision of each vernacular name [49]. We then used the Global wood density database [50] to compute simulations of mean wood gravity at the plot scale (WSG_plot_). Next, for each plot area of S_plot_ ha, we computed the above-ground biomass per hectare (AGB_plot_) using the determinist model from Eq 2.

$${AGB}_{tree}=0.0673\times {\left(WSG\times DBH^{2}\times H\right)}^{0.973}$$ (1)

$${AGB}_{plot}=\frac{\sum_{DBHclass}\sum_{trees}0.0673*{\left({WSG}_{plot}\times D_{DBHclass}^{2}\times H_{DBHclass/plot}\right)}^{0.973}}{S_{plot}}$$ (2)

AGB estimations were repeated 1,000 times for each plot in order to evaluate uncertainties due to measurement errors. We did not take the errors due to allometry (i.e. Eq 1) into account because the resulting uncertainty (though very high at the individual tree scale [47,51,52]), was expected to be cancelled out or at least drastically reduced on such large plots (as suggested by previous studies [47,51,53,54]). We verified *a posteriori* that this hypothesis was correct and that propagating all uncertainties in our tests didn’t change our results (see S3 Text). Despite the approximate measurements of forest inventories, coefficient of variation (CV) of AGB estimates at the plot scale rarely exceeded 17% for CTFT data and 10% for ONF data. So in the worst case, the confidence interval of mean AGB estimates at 95% for plots did not exceed 6 Mg.ha^-1^. Finally, in order to compare our results with previous similar studies (see Table 1), we simply used mean AGB estimates for plots and didn’t propagate uncertainty in the following tests (see S1 Dataset).

### Statistical analyses

**Fig 2:**
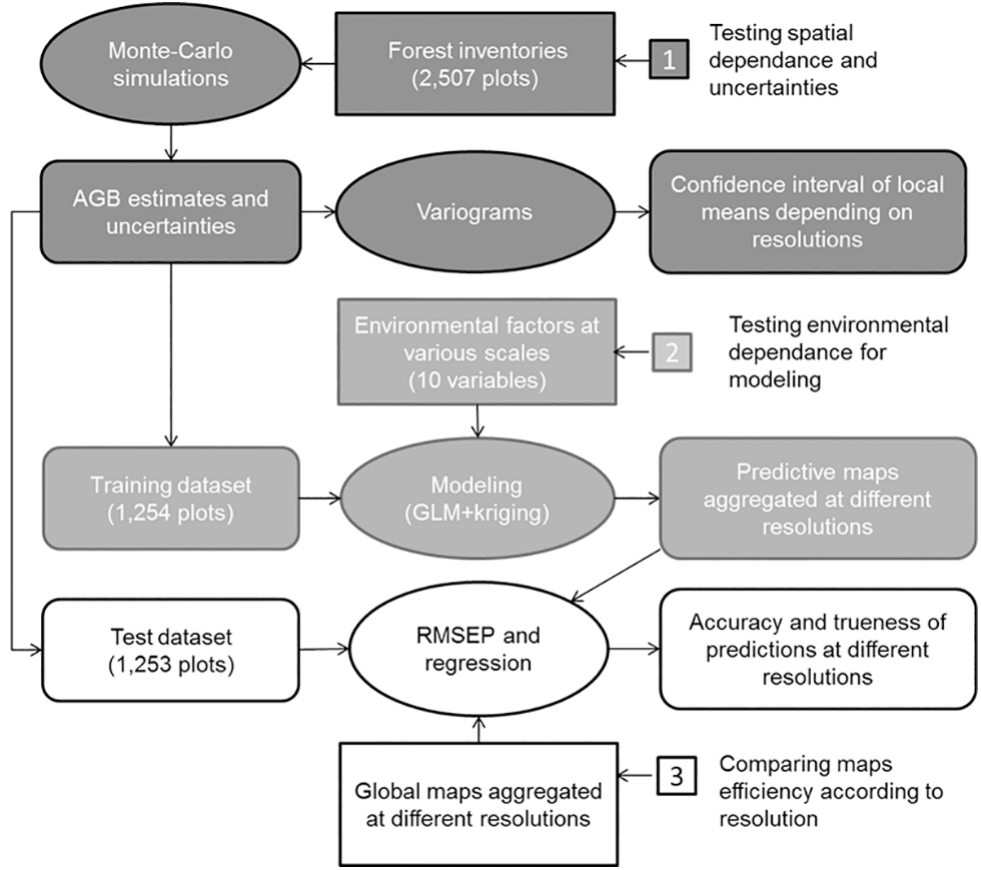
Flowchart followed for statistical analyses. The grey colours indicate the different steps of analysis. Input data are represented in rectangles, analysis in ellipse and outputs in rounded boxes.

Statistical analyses followed three steps that are summarized in Fig 2. In the first step we used all our data to produce variograms in order to examine the spatial structure of biomass and its consequences in terms of accuracy for interpolating from field data (dark grey on Fig 2). In a second step we used half of our data (training dataset) to calibrate prediction models in order to produce AGB maps at different resolutions and to evaluate the influence of environment factors on AGB variation (medium grey on Fig 2). In the last step we used the second half of our data (test dataset) to compute Residual Mean Square Error of Prediction (RMSEP) and regressions in order to test the accuracy of our maps at different resolution and to compare it with the accuracy of extant global maps (from Baccini [15] and Saatchi [16]).

#### Testing spatial dependences and uncertainties due to local variability

To detect spatial dependences in biomass distribution, we performed a variogram (semi-variance) analysis (package geoR–[55]) using all our data. Mean semi-variance was computed for 10 distance classes (i.e. limits at 500 m, 1 km, 2, 4, 8, 16, 32, 64, 120, and 240 km) and compared with the null hypothesis of an absence of spatial structure simulated by 1,000 randomizations of AGB values between plots. At any distance class L (between L_1_ km and L_2_ km), having the observed semi-variance under the random confidence interval indicated significant auto-correlation, whereas semi-variance above this confidence interval indicated significant over-dispersion.

We then assessed the implication of the spatial structure for the computation of the theoretical confidence interval of the local AGB mean. We first generated systematic grids with different cell-sizes (resolution R = 0.5 km – 1 km – 2 km – 4 km – 8 km) and selected all cells including at least three plots. This yielded 142 to 316 cell values per grid. We then computed the coefficient of variation (CV) and the 95% confidence interval of the local estimates (IC_95%_) using Eq 3, and we modelled the relationship between IC_95%_, N and R using log-log regressions.

$${IC}_{95\%}=t_{N,95}\times \frac{CV}{\surd N}$$ (3)

#### Modeling biomass with GLM and ordinary kriging

We tested general linear models (GLM) of every possible first-order combination of environmental variables (package R stats and glmulti—[56]) in order to (i) test to what extent environmental effects can explain biomass spatial variation; (ii) interpret the main significant effects; (iii) produce the most efficient predictive map. We used the Akaike Information Criterion (AIC) to select the best and most parsimonious model and verified *a priori* hypotheses: normality of the residues (Kolmogorov-Smirnov test), heteroscedasticity (Breusch-Pagan test) and independence of the residues (variogram). We tested the different terms of the model using ANOVA.

In a first step we included the type of inventory (i.e. CTFT versus ONF) in the GLM in addition to environmental factors in order to test the bias that could be introduced by joining old and new field data. The variable was selected in the best model but its effect proved to be very limited and non-significant regarding ANOVA-test (coefficient -12.7, ddl = 1, F value = 2.32, p = 0.127). We then renewed the test without this factor. The new model led to the selection of the same environmental factors than in the first test with a slight increase in AIC (11541 versus 11540) and similar predictions (no bias—R² = 0.985). As a consequence, we neglected inventory effect thereafter.

As spatial correlation was detected in the residues of GLM, we added a spatial component k(s) to the deterministic terms of the model (i.e. Eq 4 with estimated mean μ and environmental effects γ_e_ for each of the variables x_e_). We modelled k(s) by ordinary kriging as proposed by [8]. We postulated an exponential function for the covariance model (i.e. Eq 5 with τ = nugget, σ² = sill, φ = range) and fitted the terms of the model to the observed variogram of residues using ordinary weighted least squares (package R geoR–[55]).

As a result, our final model to predict AGB at the location s, noted y(s), is a kriging-regression model (KR) which took the following form:

$$y_{\left(s\right)}=\mu +\sum_{e=1}^{E}\gamma_{e}\times x_{e}(s)+k(s)$$ (4)

With the covariance model of k following the exponential form:

$$\gamma (d)=\tau +(\sigma ²-\tau )\times \left(1-\exp\left(-\frac{d}{\varphi }\right)\right)$$ (5)

We used half of our dataset to calibrate this model, systematically selecting one plot out of two (training set) and reserved the rest (test dataset) to compute the root mean square error of prediction (RMSEP) of our model in the validation step. We preferred this conservative method to more sophisticated cross-validation ones (e.g. one-out-of bag) in order to limit the risk of over fitting (e.g. [35]).

#### Mapping and comparing our model with other models at different scales

We first applied our GLM and KR models to produce maps at the finest possible resolution (30 m) on GIS. We then produced coarse-grained maps by local averaging at different resolutions (1 km, 2 km and 4 km) using aggregation processes (R-package raster [57]). We performed the same process on pan-tropical maps produced by Baccini [15] and Saatchi [16] and aligned the four maps, in order to compare their accuracy with ours at the different resolution For each resolution, we then selected cells featuring more than three validation plots from the test dataset and we computed RMSEP using the local means. We also examined the precision and trueness of the maps using respectively the correlation coefficient (R²) and slope of linear regression between the predictions and the test dataset.

Finally, to evaluate the practical relevance of the different maps at operational scales (i.e. at the scale of a forest concession, between 10,000 and 100,000 ha, or at the scale of a local forest management project, between 1,000 and 5,000 ha), we computed the same indicators (RMSEP, r², slope of regression) for the range of areas displayed by the CTFT inventory blocks (166 to 800 km²) and for the ONF inventory sites (10 to 47 km²). We supposed these areas to be within the range leading from forest concession to large REDD project areas (>100 km²) or local REDD project areas (10–50 km²). We also compared the biomass estimates obtained with our training set and those obtained with our test set for these areas to see the absolute accuracy we could expect from forest inventories at these scales.

## Results

### A high local variability and a weak spatial structure led to large uncertainties in local biomass estimates

**Fig 3:**
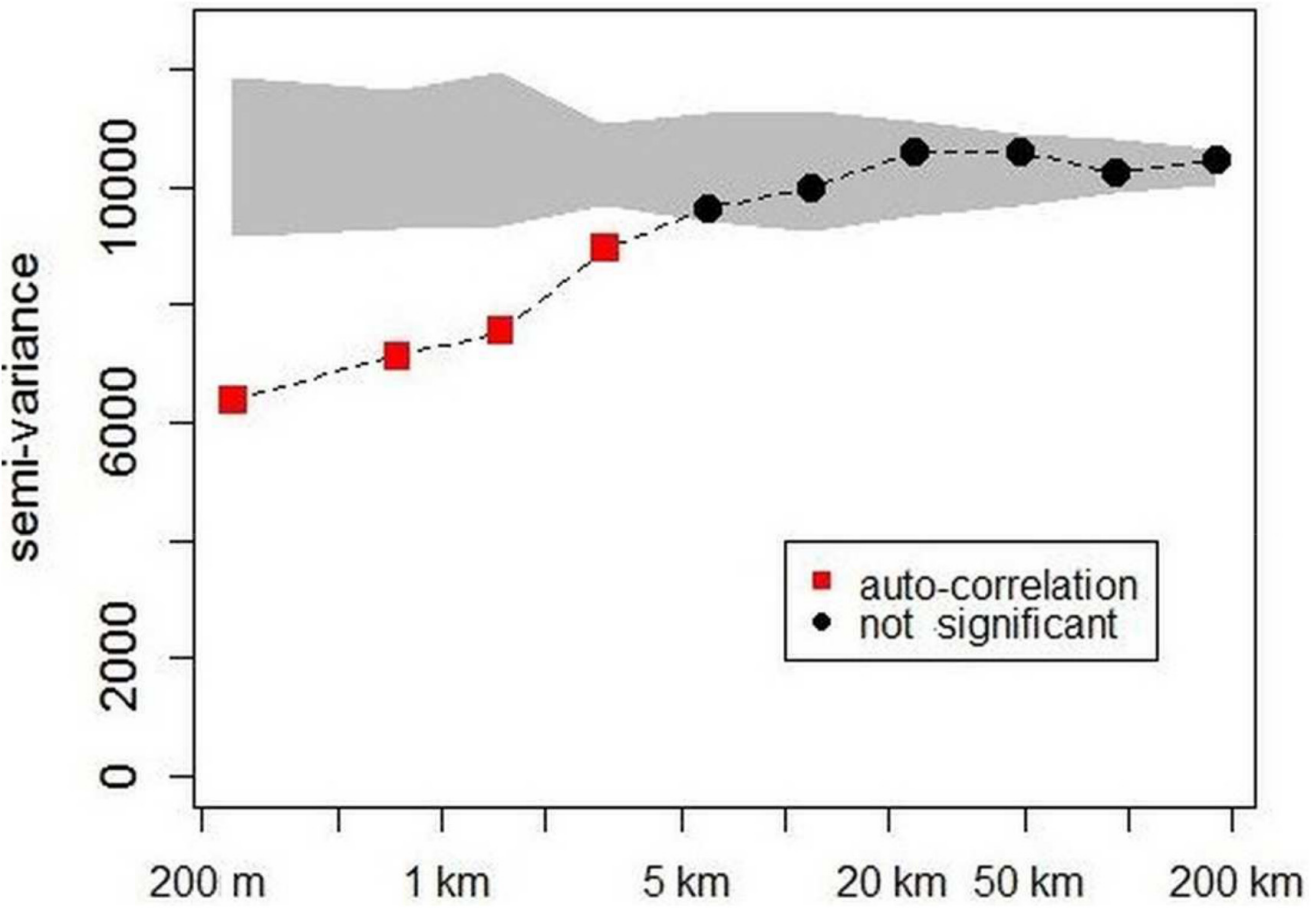
Variogram of biomass estimates from 500 m to 200 km according to distance classes. The grey shape shows the confidence interval expected for each distance class under the null hypothesis (1,000 randomizations). The red squares indicate significant auto-correlation and the black circles imply no significant correlation.

Variograms applied on the mixed datasets **(Fig 3)** showed high and significant semi-variance at the beginning of the curve (i.e. nugget of about 6,000 equivalent to a difference of about 110 Mg.ha^-1^ between neighbouring plots, located less than 500 m apart). Semi-variance increased rapidly up to 20 km and remained a fairly stable from 20 km to 200 km (i.e. sill of about 10,000 equivalent to a difference of about 140 Mg.ha^-1^) approximating the overall variance. Comparison with 1,000 randomizations indicated a significant autocorrelation for distances of less than 5 km and no other significant effect for larger distances. This shape of variogram with high nugget effect (local variability) and short range of auto-correlation corresponded to a weak spatial structure (i.e. very limited spatial dependence in biomass variation).

**Fig 4:**
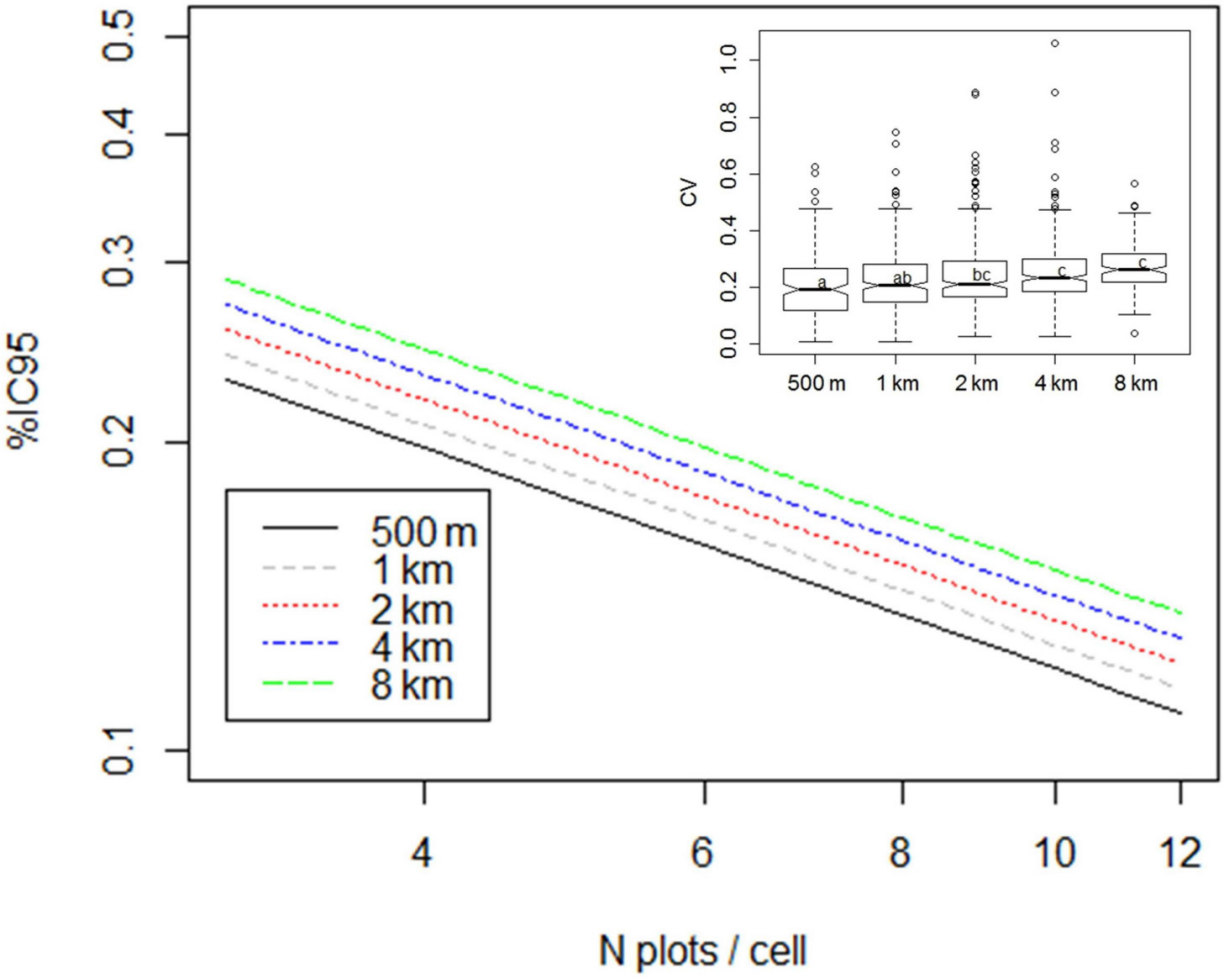
Estimation of coefficient of variation and confidence interval of local mean according to grid resolution. Inset boxplot shows CV of local biomass estimates according to grid resolution. Main part shows the fitted values confidence interval of the local mean according to the model log (IC_95%_) = -0.59277–0.59128 * log (N) + 0.04988 * log (R) from N = 3 to 20 and R = 0.5 km to 8 km.

In accordance with the previous variogram, CV of local AGB estimates had a quite high mean value (about 21%) when computed on the 0.5 km resolution grid. It increased very slowly, up to 27%, when we increased the size of the grid cells from 1 km to 8 km **(Fig 4)**. Modelling IC_95%_ (relative confidence interval of local AGB estimates) from the number of field plots (N) and grid resolution (R), led to the log-log model (r² = 0.82, F = 232, DF = 99, p<0.001, AIC = -24):

$$\log\left({\mathrm{IC}}_{95\%}\right)=-0.59277-0.59128\times \log(N)+0.04988\times \log\left(R\right)$$ (6)

We observed that %IC_95_ decreased mainly with an increase in the number of calibration points and increased slowly with resolution **(Fig 4)**. As a result, for the same sampling density, the uncertainty of local biomass estimates was halved when the cell size was doubled (e.g. IC_95%_ = 24% for three plots on average in 1 km-cells and IC_95%_ = 13% for 12 plots in 2 km-cells).

### The environment accounts for a significant but minor part of spatial variation in biomass

**Fig 5:**
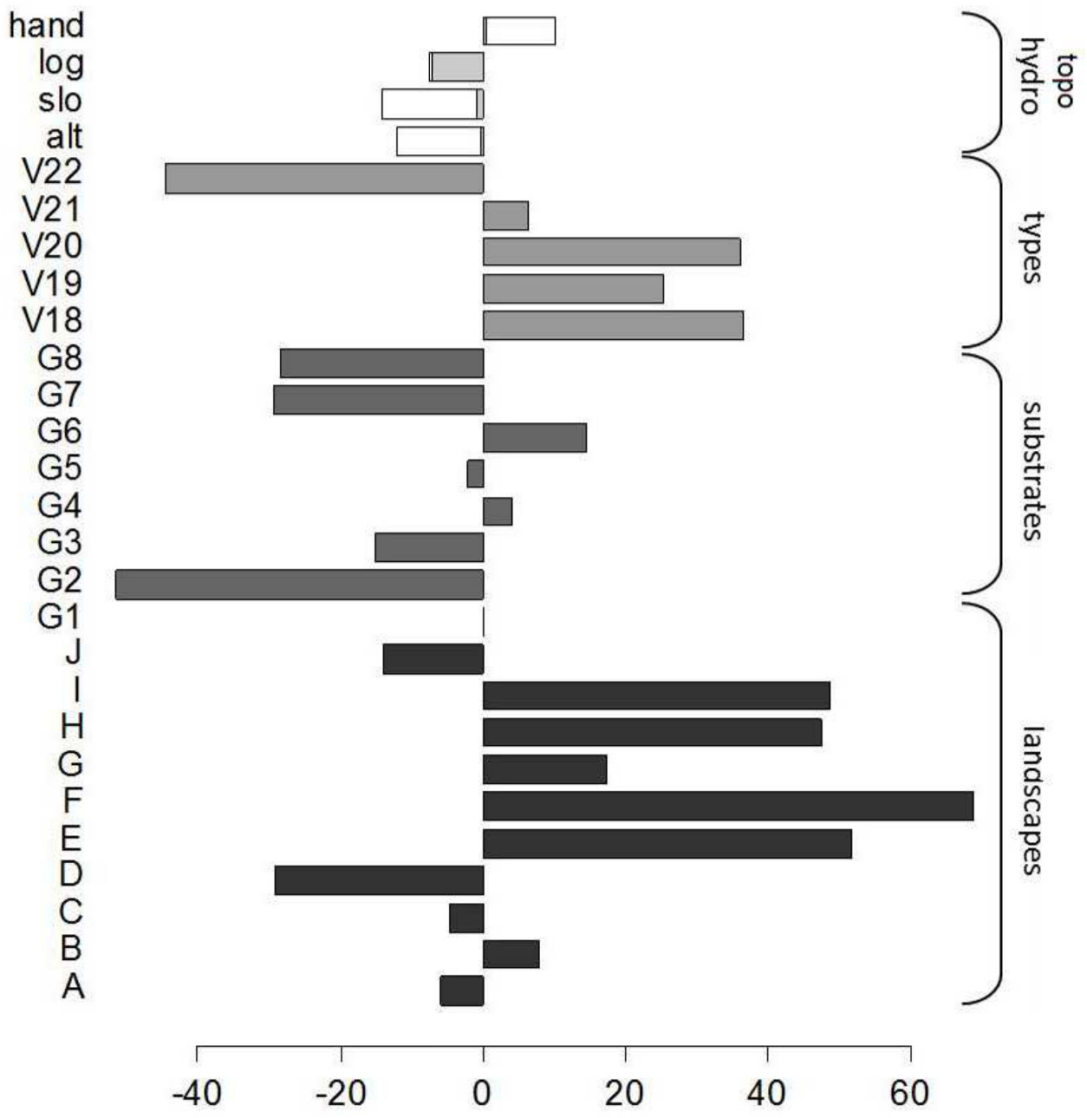
Coefficients of the selected GLM that predict biomass from environmental variables. Grey bars and brackets indicate groups of modalities related to the different categorical variables. For continuous topographical and hydrographical variables (HAND, LOG, SLOpe and ALTitude) the coefficient value is multiplied by the mean of the variable.

The best GLM selected to predict biomass variation with environmental variables explained a small but significant proportion of variance when fitted on the training set (R² = 0.09, DF = 1228, p<0.001, AIC = 11 541 with intercept = 0). Geomorphological landforms, the dry season index and annual rainfall were excluded from the model **(Fig 5)**. Geomorphological landscapes had the strongest effect on biomass (F = 6.0664, DF = 10, p<0.001) and displayed marked contrasts at large scales between (i) on the one hand, regions dominated by mountains (H), plateaus (E,F,G), or smoothed multi-convex landscapes (I) with high biomass; and (ii) on the other hand, plains (A), valleys (C), multi-concave (D) and marked multi-convex landscapes (B,J) with low biomass. Low HAND (height above the nearest drainage) and high LOG (logarithm of basin area), which mainly point to seasonally flooded forests, were also highly influential at the local scale with a significant negative effect (respectively F = 11.3495, p<0.001 and F = 9.9309, p<0.01). GEOL (Geology) had a significant but limited effect driven by the “dykes” category (G2), which corresponded to an extremely hard localized substrate with significantly lower biomass (F = 2.5477, DF = 7, p<0.05). Similarly, VEGET (vegetation type) had only a slight effect mainly driven by type 22 which exhibited very low biomass (F = 2.8287, DF = 5, p<0.05) and corresponded to “open forests mixed with palm forests”, mainly located in the southern part of French Guiana. Altitude and slope had the weakest effects (respectively F = 3.5036, p<0.1 and F = 2.5405, p = 0.111).

**Fig 6:**
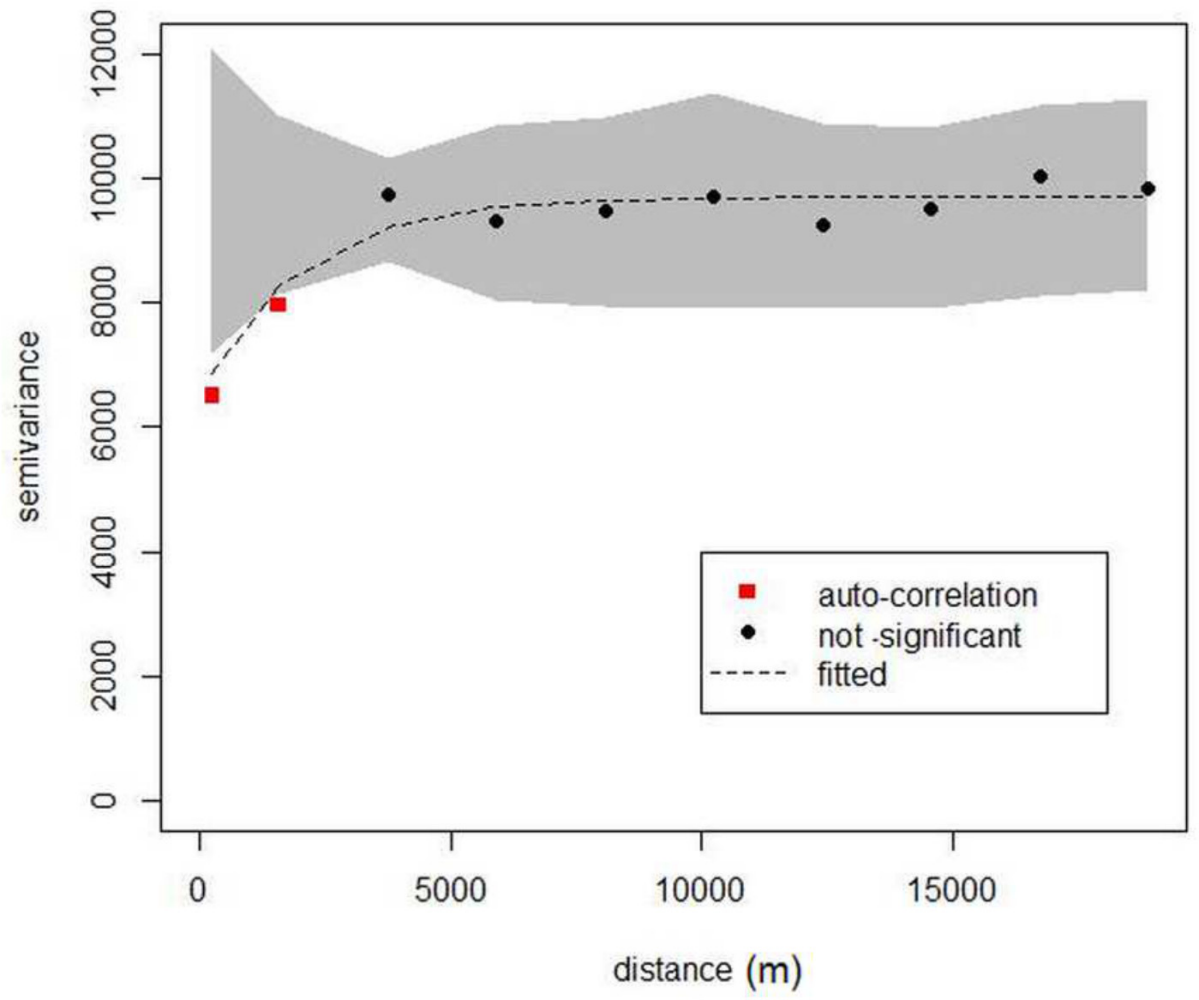
Variogram of GLM residuals from 500 m to 200 km according to distance classes. The grey shape indicates values expected under the null hypothesis of absence of spatial structure (1,000 randomizations). The red squares indicate significant auto-correlation and the black circles no significant structure. The dashed line represents the fitted exponential model used to predict the spatial error term.

The variogram computed on the residuals of the model still showed significant spatial dependence for distances of less than 2 km **(Fig 6)**. We modelled this residual structure by kriging-regression (KR) using an exponential covariance function (τ = 6500, σ = 3205, φ = 1991 –fixed kappa = 0.5).

**Fig 7:**
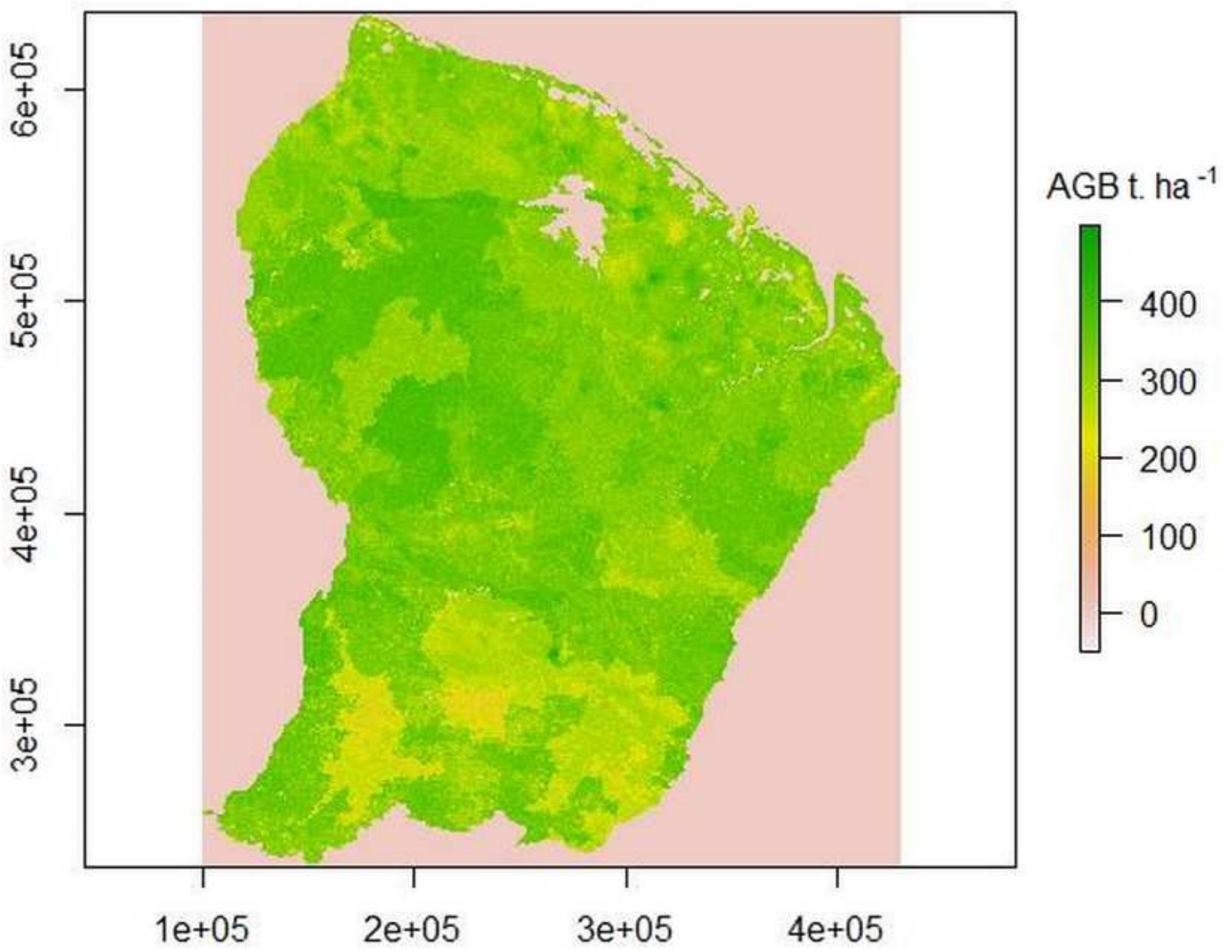
Map of AGB (Mg.ha^-1^) in French Guiana based on the complete model (KR). Local darker or paler areas correspond to spatial error terms that can be modelled only within a short distance of the calibration plots and are null over most of the area.

As a result, when we applied the GLM (deterministic part of the calibrated model) to the test set, we obtained a quite large RMSEP of 99t MS/ha and a poor adjustment (r² = 0.04, DF = 1251, p<0.001, slope = 0.063). When we used the complete model KR, we increased the accuracy of the model (RMSEP = 90t MS/ha) with a better adjustment (r² = 0.20, DF = 1251, p<0.001, slope = 0.208). However, as the practical range of auto-correlation used in kriging is short, it did not enable residual error to be predicted beyond a distance of 7 km around the sampling locations. Consequently, model accuracy was only actually improved by the spatial component in a small part of the territory located near the sampling areas used for calibration **(Fig 7)**.

### Coarse-graining improved the accuracy of maps

The pan-tropical maps at their original resolution (i.e. 1km) were poorly correlated with the test dataset (RMSEP > 80, R² = 0.02 and slope ≤0.1) whereas the accuracy of our complete model (i.e. KR) was largely improved at this resolution (RMSEP = 63, R² = 0.35 and slope = 0.55).

**Fig 8:**
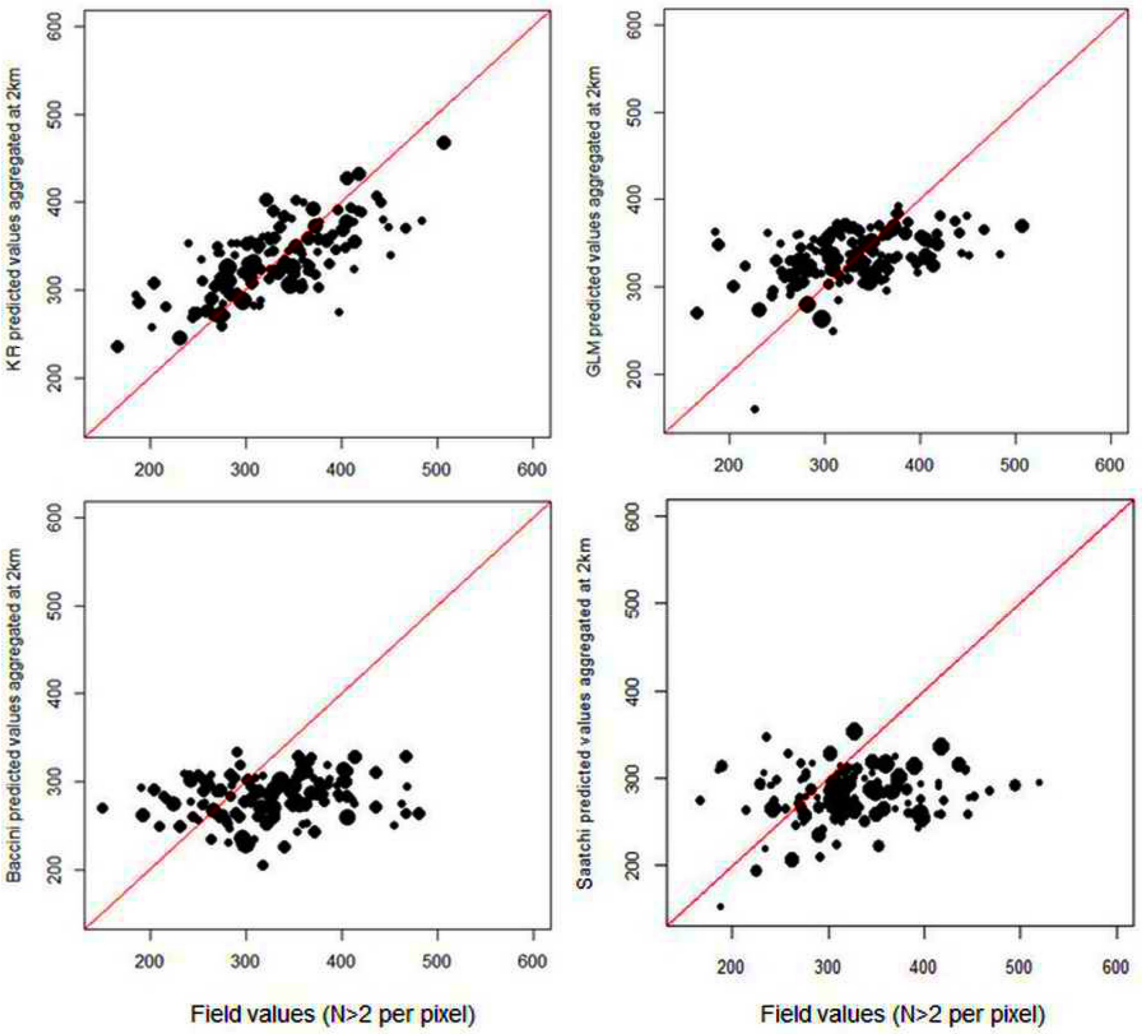
Comparison of AGB values predicted by the different maps at 2-km resolution with test dataset. Aboveground biomass (AGB) means at cell level for the test set are compared to the values predicted by the different maps at 2-km resolution: from the top left to the bottom right—KR, GLM, Baccini [15] and Saatchi [16]. The red line indicates the 1:1 relationship (expected slope). The size of the circles indicates the number of plots for each cell in the test set (from 3 for the smallest to 12 for the biggest).

The same results were obtained with 2-km resolution cells **(Table 3 and Fig 8)**. Thanks to coarse-graining, our models were more accurate than at the finest resolution (RMSEP was reduced by 25% for GLM and 30% for KR compared with 1-km resolution), with better precision (r² = 0.19 and 0.48 for GLM and KR respectively with p<0.001 in both cases). However the regression slopes kept the same value as at 1-km resolution (i.e. about 0.2 for GLM and about 0.5 for KR), indicating a dilution bias effect that could not be reduced. As a result, bias was zero for mean values but systematically negative for the highest values and positive for the lowest values (Fig 8). At and above 4-km resolution, all statistics of adjustment were degraded or saturated on all maps (RMSEP, R² and the same or worse slope than previously).

**Table 3:** Accuracy of the different maps for different cell resolutions.

Resolution	Map	RMSEP^a^	R²	Slope^a^
1 km	GLM	74	0.09**	0.21
	**KR**	**63**	0.35***	**0.55**
	Baccini [15]	85	0.02^ns^	0.08
	Saatchi [16]	91	0.02^ns^	0.10
2 km	GLM	58	0.19***	0.21
	**KR**	**47**	0.48***	**0.49**
	Baccini [15]	80	0.02 ^ns^	0.06
	Saatchi [16]	85	0.01^ns^	0.06
4 km	GLM	59	0.15***	0.14
	**KR**	**46**	0.49***	**0.40**
	Baccini [15]	85	0.02 ^ns^	0.04
	Saatchi [16]	94	<0.01 ^ns^	-0.02

^a^ The root mean square error of prediction (RMSEP) indicates the overall accuracy, the R² indicates the precision, and the slope indicates the trueness of the models. The significance of the adjusted-R² was tested with a F test (*** p<0.001, ** p<0.01, * p<0.05, ns = non-significant)

### Satisfactory accuracy can be achieve for REDD+ operational scales

The comparison of our test dataset and training dataset showed that forest inventories with a sampling rate of between 0.1 and 0.5% estimated biomass with an accuracy <10% for large blocks (>100 km²) and for a large majority of 10 to 50-km² sites (respectively RMSE = 10 and 26 Mg.ha^-1^ –**Table 4**). At these operational scales, our complete KR model estimated total biomass with good accuracy (RMSE = 31 Mg.ha^-1^ for ONF sites and 18 Mg.ha^-1^ for CTFT blocks), whereas simple GLM led to larger errors (Table 4). AGB estimates obtained with pan-tropical maps were absolutely not correlated with our test dataset, with the exception of Baccini’s map for areas between 10–50 km² (r² = 0.15 –p<0.05).

**Table 4:** Evaluation of the accuracy of the different models at operational scales.

Scale	Estimates	RMSEP^a^	R²	Slope^a^
Small project, production units (10–50 km²)	**training set**	**26**	0.67***	**0.868**
	GLM	40	0.32***	0.339
	**KR**	**31**	0.64***	**0.551**
	Baccini [15]	61	0.15*	0.155
	Saatchi [16]	70	<0.01 ^ns^	0.079
Large project, concessions (>100 km²)	**training set**	**10**	0.93***	**0.885**
	GLM	40	0.59**	0.103
	**KR**	**18**	0.90***	**0.390**
	Baccini [15]	74	<0.01 ^ns^	-0.037
	Saatchi [16]	56	0.07^ns^	-0.118

^a^ The root mean square error of prediction (RMSEP) indicates the overall accuracy, the R² indicates the precision, and the slope indicates the trueness of the models. The significance of the adjusted-R² was tested with a F test (*** p<0.001, ** p<0.01, * p<0.05, ns = non-significant)

## Discussion

### High local variability limits the accuracy of biomass maps

The spatial structure of aboveground biomass (AGB) has already been shown to be highly variable at spatial resolutions less than 250 m when measured in forest plots ranging from 6 to 50 ha in size [19,21]. The high local variability can be explained by gap-phase dynamics which create a mosaic of eco-units [19,58]. This local variability implies that significant “dilution bias” will occur when small plots (i.e. substantially less than 0.5 ha) are used to calibrate models to be applied over larger areas. Upscaling leads to underestimation of the spatial variance of AGB in the models [21]. Our study proves that AGB variability is already very high at spatial resolutions of between 200 and 500 m and increases even more at more than 5 km (Fig 3) whereas one might expect that this effect, which is linked to forest dynamics, would be mitigated over large areas (i.e. >1 km²). As a result, “dilution bias” is likely to occur even when large forest plots i.e. ca. 1 ha, are used to calibrate models with standard satellite remote-sensing data, as it is widely recommended [21]. Dilution bias is also to be expected when a few small footprints covering areas of the same order of magnitude as field plots are used as sampling units to calibrate larger footprints (e.g. airborne LiDAR transects or GLASS footprints used to calibrate MODIS or LANDSAT pixels). This helps explain why pan-tropical maps, which are based on double upscaling (from field to high resolution RS and then to medium resolution RS) fail to capture the forest spatial variability of AGB with acceptable accuracy [7].

The high local variability measured in our forest inventory plots (average standard deviation above 100 Mg.ha^-1^), cannot be the result of unexpected noise in the inventory data, because (i) the sizes of our plots, between 0.4 and 0.5 ha, are sufficient to limit in-and-out effects for large trees [20]; (ii) uncertainties and local variability computed at the plot scale are consistent with previous studies [59]; (iii) the local CV measured within 500 m-resolution cells was of the same order of magnitude as reported for 200 m in other studies based on permanent plots in a tropical forest [21]. Obviously, the heterogeneity between the different sources of data has been correctly controlled since no important bias due to inventory was detected in the analysis. This is in accordance with previous study which detected only a slight increase in AGB during the past decade in our region (<0.9 Mg.ha^-1^.y^-1^) with no statistical significance [39]. Moreover, we verified that errors due to allometry and measurements led to very limited uncertainties (i.e. <15%).

We therefore conclude that this high local variance is not an artefact, but is likely due to the heterogeneous forest structure [22], which in turn, could be explained by (i) local topographic and hydrologic effects (included in our GLM with altitude, HAND and basin area); (ii) the distribution of big trees which often exhibit aggregative patterns driven by population dynamics and species dispersion in the long term [60]; (iii) large-scale natural forest disturbances such as landslides or blowdown, even if gaps of more than 1 ha have been shown to be quite rare in northern Amazonia [61].

Environmental factors are partly efficient to capture this structural variability, rather due to stochastic processes. In fact, the deterministic part of our model explained a modest part of the variance. However, our model was able to detect contrasts along the waterlogging gradient on the topographic sequence [62,63] as well as large scale variations at the landscape scale [64]. Nevertheless, as shown in previous studies, even in the case of strong environmental contrasts monitored at fine scale (e.g. waterlogged vs. never flooded locations or white sand vs. other terra firme forests) pure environmental effects only explain a small fraction of variations in AGB and interact largely with more important structural effects [65].

The important consequence of major variations in AGB at short distance, as evidenced by the present study, which corroborates another recent one [22], is that any statistical interpolation between scare field reference points will remain imprecise whatever the accuracy of the field measurements. The only way to enhance precision is to combine geo-statistical interpolation (e.g. kriging) with the most relevant spatialized environmental information, as exemplified in the present study. Reciprocally, incorporating a spatial component in the biomass model is an effective way to mitigate the problem of weakly environmentally structured variation in AGB and to substantially improve its efficiency [8,35]. However, we showed that this improvement is limited to a short distance around the reference points, because of the short autocorrelation range (i.e. a few kilometres).

### Sampling design and spatial resolution have to be adapted to capture AGB spatial structure

The most cost-effective way to capture and control the local variability is to adapt the resolution of the output AGB map to average out local variability. In other words, a trade-off needs to be found between spatial resolution and effective accuracy. On the one hand, reducing output resolution minimizes local variance, but on the other hand, enlarging resolution helps calibrate the model with more precision by multiplying the number of field plots per calibration-cell, which is the most efficient way to reduce the confidence interval of local estimates **(Fig 4)**. In our case, a 2-km resolution (4 km²) appears to be the optimal trade-off between a minimum RMSEP and maximum adjustment (r² and slope approaching 1—see **Fig 8 and Table 2**) for calibration. From a practical point of view, this means that AGB maps should not target a resolution (output cell size) of less than few kilometres otherwise there is a risk of very high uncertainty at individual cell level that will result in a poor calibration step.

Moreover, our results suggest that regular systematic sampling is not the best way to calibrate AGB maps. For instance, to accurately calibrate a model with less than an average of 5% error at a 2-km resolution, more than 75 plots are necessary per calibration cell, 20 plots for 10% and 5 plots for 20%, which corresponds to a sampling rate of 8%, 2% and 0.5% respectively. Such very high sampling rates required to obtain accurate local means for calibration, are not economically sustainable for very large areas [22]. A multi-scale stratified sampling design appears to be a better way of ensuring both a sufficient sampling rate for local calibration and sufficient general coverage to account for the broad-scale patterns of variation in AGB.

Achieving “wall-to-wall” LiDAR over large areas is a reliable alternative way to address these methodological limits in the medium term [66]. However, a significant gap still exists between the complexity and cost of this method on the one hand, and the actual capacity of developing countries to implement it on large areas on the other hand [3]. Here, using large forest inventories even with a quite low sampling rate (i.e. about 0.01% for the whole territory and from 0.1 to 0.4% locally) we succeeded in producing AGB maps with acceptable RMSEP ranging from 47 to 58 Mg.ha^-1^ (i.e. 22 to 28 MgC.ha^-1^ that to say, a relative error of 15% to 20%) at a 2-km resolution depending on the distance from the nearest sampled area. As a matter of fact, for operational management of forest resources at local to national scales (e.g. evaluation of biomass for a REDD+ project or LULUCF national monitoring) rapid forest inventories with suitable design may suffice to produce accurate estimates at the appropriate resolution for an output map.

### Combining remote-sensing and large scale forest inventories can improve the accuracy of biomass maps

Our review of literature focusing on “biomass mapping in tropical forest”, shows that RMSE hardly reach 75Mg.ha^-1^ in old-growth forests (i.e. a relative error of about 20%), for a 1-km resolution or less **(Table 1)**. The performance obtained with our forest inventories, even at 1-km resolution, is better than the majority of biomass maps in the literature [7,15,16,31–33]. Most studies which report RMSE lower than 75Mg.ha^-1^, include different vegetation types such as savannahs, opened forest or young plantation, thus mechanically reducing absolute RMSE (e.g. [28,29] in **Table 1**), or having to deduce AGB from rough DBH-based allometries that do not account for variations in wood density and height within the forest and hence artificially reduce actual variance (e.g. [8,27,28] in **Table 1**). The most efficient AGB maps are based on the “wall-to-wall” LiDAR method, but remain very limited in extent, i.e. to only a few km² [28,34]. However, a better performance was reported for two maps covering larger areas with RMSE down to 55 Mg.ha^-1^ (i.e. 27 MgC.ha^-1^) at 100-m resolution, using LiDAR to calibrate AGB estimates for forest strata based on LANDSAT and SRTM data [30,35]. These two studies concluded that the final upscaling step is critical to ensure the efficiency of biomass mapping and to better capture spatial autocorrelation to fully transform the potential display by LiDAR altimetry into local AGB predictions at a broad scale.

Our results suggest two ways of improving this upscaling process. First, holistic and multi-scale geomorphological maps can provide an efficient basis for preliminary forest stratification to guide LiDAR acquisition as well as the sampling of field data. This preliminary stratification step is too often limited to *a priori* expert-based stratification (e.g. altitude threshold, catchment basin delimitation, etc.). A formalized geomorphological analysis based on full-resolution SRTM (as done here) would help define precise and objective relief stratification (e.g. a plateau vs. a multi-convex landscape) while subjectivity can be controlled by using image analysis techniques to characterize and classify landforms (see for instance [43,67,68]). This would also make it possible to delimit local habitats (i.e. terra firme vs. seasonally flooded forests), thus reducing within-strata variability [38]. Second, our results demonstrate that field plots in forest inventories are a reliable source of accurate field measurements of AGB. Such field data can thus be used to locally calibrate and validate LiDAR allometry (leading from canopy altimetry metrics towards AGB) as well as any kind of biophysical information derived from remote-sensing data of sufficient resolution that can contribute to AGB mapping (e.g. canopy texture as exemplified in [31,69]). Future progress will indeed rely on smart sampling and upscaling schemes from highly informative (regarding forest structure and biomass mapping) albeit costly data sources such as field inventories and small footprint LiDAR flight lines, to satellite remote sensing information of higher affordability and replicability.

Given such strong local variations in AGB along with short range autocorrelation, the upscaling scheme is indeed critical, and understanding the relationships between variations in above-ground biomass and landscape patterns is a promising way to base the upscaling process on broad scale drivers of AGB variations via variables which can conceptually and statistically be derived from worldwide databases and satellite remote sensing. Combining forest inventories along transects with LiDAR flight lines could be an efficient way to improve the global coverage of AGB maps of tropical forests while maximizing field datasets and capturing cryptic regional variations (e.g. patterns in wood density, changes in allometry between forest types) that are easily overlooked without an extensive integrated sampling strategy.

## Supporting Information

S1 Dataset Aboveground biomass estimates for the 2507 plots. The file provides 1,000 estimates of AGB for the 2507 plots and specifies the geographic coordinates of the centre of the plot (WSG84UTM22N) and the area of the plot (ha).
(XLSX)

S1 Text Details about field data. (DOCX)

S2 Text H:D sub-model used in allometry. (DOCX)

S3 Text Propagation of errors due to allometry. (DOCX)
