# Supplementary material for: Spatial Structure of Above-Ground Biomass Limits Accuracy of Carbon Mapping in Rainforest but Large Scale Forest Inventories Can Help to Overcome
Source: PLoS One. 2015 Sep 24;10(9):e0138456. doi: 10.1371/journal.pone.0138456 (PMC4581701; doi:10.1371/journal.pone.0138456)
Supplement: S1 Text — (DOCX) [file pone.0138456.s002.docx]

## SI1: Details about field data

#### CTFT inventory:

The first inventory was done by CTFT (Centre Technique Forestier Tropical) between 1974 and 1976 on 14 blocks covering a total of 547,000 ha in the northern part of the French Guiana. It was based on 0.5-ha plots (250 m x 20 m) implanted on systematic grids with different intervals with a sampling rate that varied between 0.2 and 0.8%. Trees with DBH above 10, 15 or 40 cm were counted and identified using vernacular names (i.e. local common names consistently used by tree spotters). DBH was recorded by 10 cm class (i.e. [10; 20 cm [, [20; 30 cm [and so on). Data from 10 blocks were scanned between 2006 and 2010 and positioned on GIS using original maps and precise field descriptions recorded in field notebooks (rivers, slope, exposure, and distances). By comparing theoretical positions (on old maps) with actual positions on GIS (deduced from field notes), we estimated the spatial accuracy to be less than 50 m for 71% of the plots, between 50 to 100 m for 20% of the plots, and more than 100 m for 9%. Plot areas were corrected using the slopes and lengths noted in the field-books. We excluded plots with partial inventories (pre-census DBH equal to 40 cm) or belonging to several forest types (i.e. mixing terra firme and seasonally flooded forest). We also eliminated all plots located in areas affected by forest harvesting or gold mining between 1974 and 2007, using GIS information provided by forest managers (Fig.1 in main article). The final dataset corresponded to 126,880 trees with DBH ≥20 cm in 1,172 plots 0.5 ha in size.

#### ONF inventory:

The second inventory was done between 2006 and 2013 on behalf of ONF (“Office National des Forêts”: the French national forest agency) to complete regional coverage and better sample environmental variability. Thirty three sites were selected mostly in the south and east of French Guiana to cover the geological and climatic conditions poorly sampled by the former CTFT inventories. Two to four 2.5 to 3-km long transects were established at each site according to the different orientations to optimally sample the local environmental variability. A total of 111 transects were established. Each 20-m wide transect was divided into 100 m segments (i.e. 0.2-ha plots) giving a total of 3,132 basic sampling units. All the plots were geo-referenced using a GPS receiver and delineated in the field using a laser. We also measured slopes and lengths to correct areas. DBH were denoted by 5 cm class above 20 cm (i.e. [17.5; 22.5cm [, [22.5; 27.5cm [, and so on). Inventoried trees were identified using vernacular names. In order to homogenize plot size with the CTFT inventory, neighbouring plots with the same environment (geology, geomorphology, vegetation) and of the same forest type (i.e. entirely terra firme or seasonally flooded forest) were lumped two-by-two. Heterogeneous plots were excluded, giving a total of 1,335 0.4-ha plots and a total of 83,075 trees (DBH ≥20 cm). All samples made in the National Park protected areas had been approved by the Scientific Committee of the Park and by the Administration council that include local communities representation.

#### Comparison of uncertainties and distribution:

**
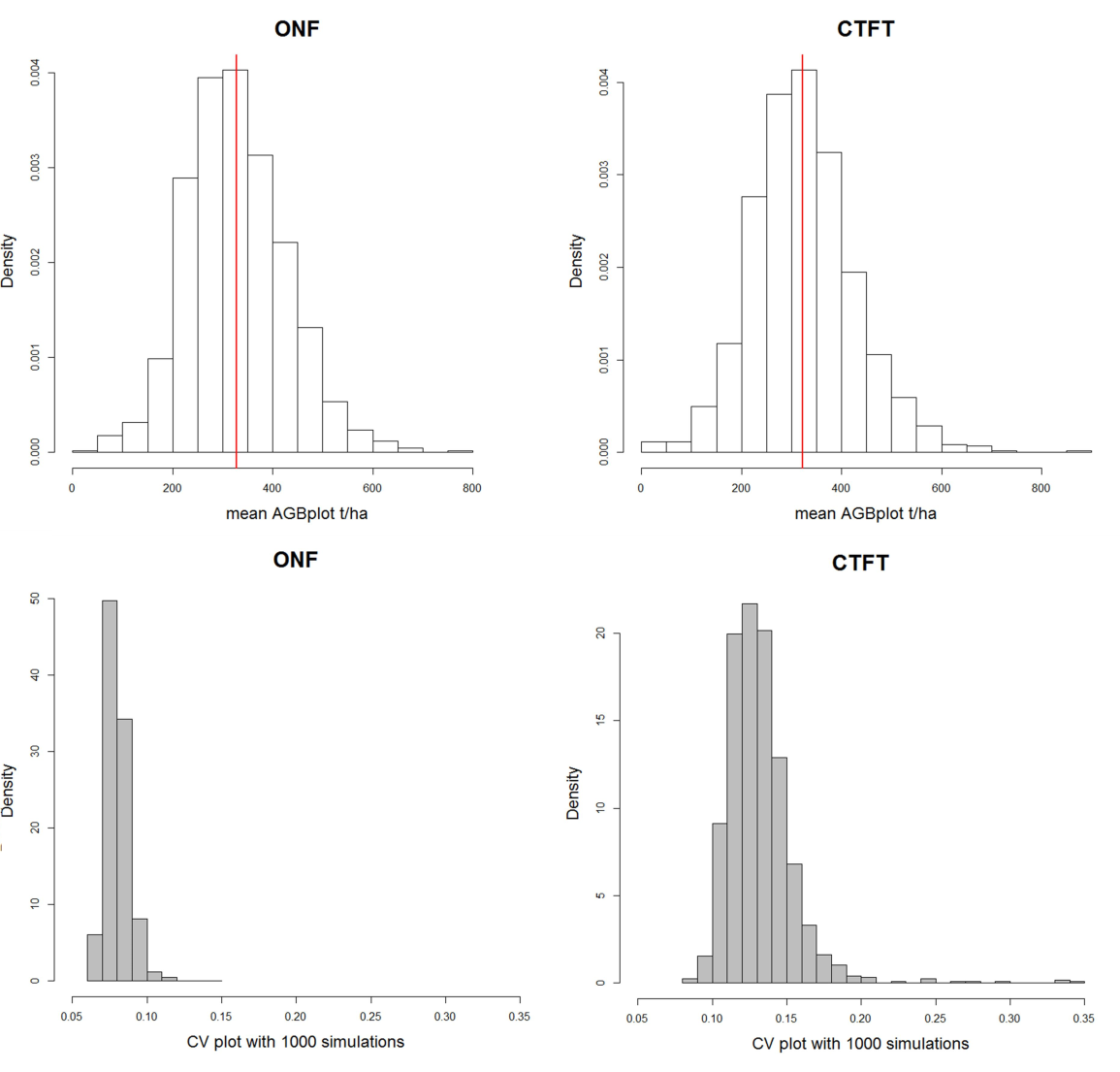
**

**Figure S1. Distribution of mean aboveground biomass (AGB) per plot (on the top) and coefficient of variation of AGB per plot (at the bottom) for the ONF inventories (left) and CTFT inventories (right)**

AGB estimates at the plot scale appeared to be less accurate using the oldest inventory. In fact, the CV of the 1000 simulations showed different distributions for the two inventories with a significantly higher mean and larger variance for CTFT inventories than for recent ONF inventories (CV=7% for ONF and 12% for CTFT, Kolmogorov-Smirnov test D=0.6593, p<0.001). However we did not detect any significant differences between the distributions of AGB estimates provided by the two campaigns (Fig. S1) despite the long-time interval between the two campaigns and the difference in field methods (Kolmogorov-Smirnov test, D = 0.0299, p-value = 0.631). As a consequence, striking similarity of the distributions allowed us to mix the two sets of data even if CTFT inventories, using wider DBH class and less accurate vernacular nomenclature, displayed larger uncertainties at the plot scale. However, at plot scale, CV rarely exceeded 17% for CTFT data (10% for ONF) so in the worst case, the confidence interval of mean AGB estimates at 95% at the plot scale did not exceed 6t.ha^-1^.
