# Supplementary material for: Spatial Structure of Above-Ground Biomass Limits Accuracy of Carbon Mapping in Rainforest but Large Scale Forest Inventories Can Help to Overcome
Source: PLoS One. 2015 Sep 24;10(9):e0138456. doi: 10.1371/journal.pone.0138456 (PMC4581701; doi:10.1371/journal.pone.0138456)
Supplement: S2 Text — (DOCX) [file pone.0138456.s003.docx]

## S2: Details about H:D sub-model used in biomass allometry:

As only DBH was measured in these inventories, tree height values were sampled from a local H:D allometry based on Michaelis-Menten model (eqn. SI1).

H_i_=(α×DBH_i_)/(γ+DBH_i_ )×ε_i_, ε_i_ ~ N(0,σ² )

This model was used for height-diameter models in French Guiana [[38](#_ENREF_38)]. To account for the spatial variability of the height-diameter relationship, the coefficients α (asymptote) and β=α/γ (slope at the beginning) were replaced by linear combinations of two stand structure variables (i.e. the proportion of small trees with DBH<40cm and the proportions of big trees with DBH≥60cm). The height-diameter model was calibrated with a Bayesian method using data collected during previous projects (9 one-hectare forest plots and 42 half-hectare forest plots [[52](#_ENREF_52)]). One thousand simulations of α and β (i.e. a combination of 3 coefficients for each) were sampled from their posterior distribution. The proportions of small and tall trees were computed for each plot such that specific height distribution could be simulated for each DBH class in each plot.


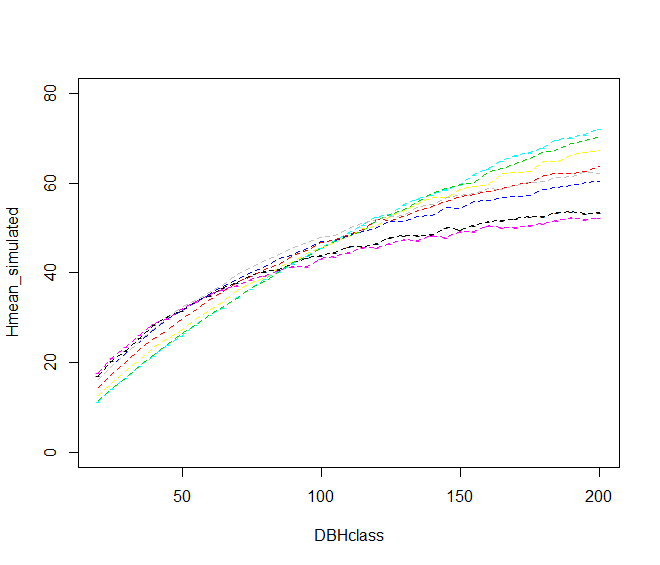


**Figure S2: Examples of H:D allometries simulated for different stand structures. Small trees (i.e. DBH<40cm) are taller when they are dominant in a plot (e.g. pink and black curves) and shorter when big trees are dominant (e.g. red and yellow curves). Conversely, the more big trees there are in the plot, the taller they will be (e.g. green and pale blue curves).**
