# Supplementary material for: Spatial Structure of Above-Ground Biomass Limits Accuracy of Carbon Mapping in Rainforest but Large Scale Forest Inventories Can Help to Overcome
Source: PLoS One. 2015 Sep 24;10(9):e0138456. doi: 10.1371/journal.pone.0138456 (PMC4581701; doi:10.1371/journal.pone.0138456)
Supplement: S3 Text — (DOCX) [file pone.0138456.s004.docx]

## S3: Propagating errors due to allometry

In the main article we already computed the uncertainty due to measurement errors and proved that they were rather limited (mean Coefficient of Variation from 1000 simulations = 7% for the recent inventory campaign and 12% for the oldest one). Chen [51] has shown that another type of error may be important, namely the error due to the allometry. In our article, following Chave and colleagues comments [47, 53] we assumed that this term of error due to the allometry, which is important at the individual tree scale, was negligible at the plot scale. To verify it actually was, we tested this hypothesis by introducing a random error following the distribution of errors given by Chave et al [47] for their model:

AGB= err*(0.0673*DBH²*H*WSG)^0.973^ with err∈N(0,0.357)

We then simulated 1000 AGB estimates per plot including a random value of the individual allometric error term in addition to simulated measurement errors for each tree.

In doing so, mean CV increased from 7% (without allometry error) to 12% (with allometry error) for ONF plots and from 12% to 14% for CTFT plots (Figure S3-1). Mean estimates for plots increased slightly (+3% for ONF and +1% for CTFT) that is an AGB mean of 329 Mg.ha^-1^ instead of 325 Mg.ha^-1^ (Figure S3-2). As a result, uncertainties due to allometry errors proved to be minor in comparison with measurement errors. It is in accordance with Chave comments [47, 53] and Chen analyses [51] from which we could expect about 10% of error regarding the surface of our plots.

As a result, because our plots are quite large (0.4-0.5ha) and because the measures are approximate in forest inventory data, the uncertainty due to the allometry error is less important than the one due to measurement errors in our study. However, to verify that our results were robust and not influenced by the uncertainty we propagated these errors in our spatial tests and GLM. For the analysis of spatial structure, we computed variograms for the first 100 simulations. All variograms showed similar patterns (Figure S3-3) with quite the same nugget effect (between 6500 and 7500) and little divergence between simulations on the first 5 kilometers. Variogram computation were applied on the AGB means including the allometry error and showed little differences with the first analysis: the nugget effect increased slightly and the sill increased from 5km to 10km. As a result, the analysis of spatial structure of AGB was not affected by the allometry error: AGB still show a weak spatial structure i.e. large local variance and very gradual increase of variance with the distance and auto-correlation limited to a few kilometers.


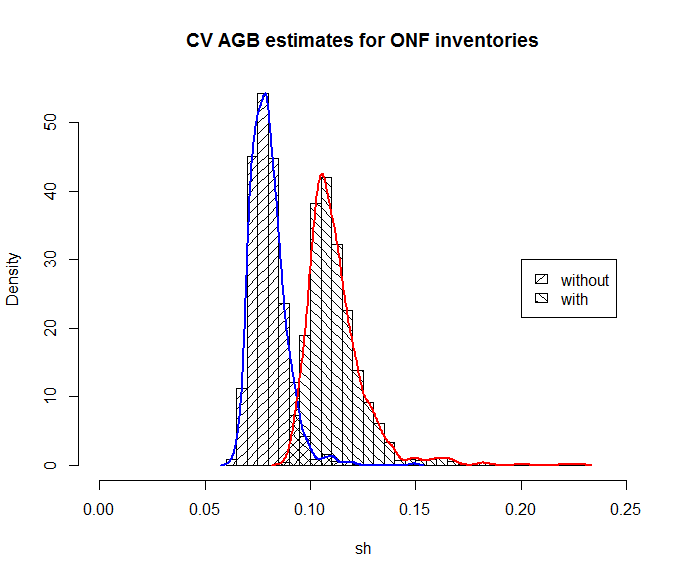

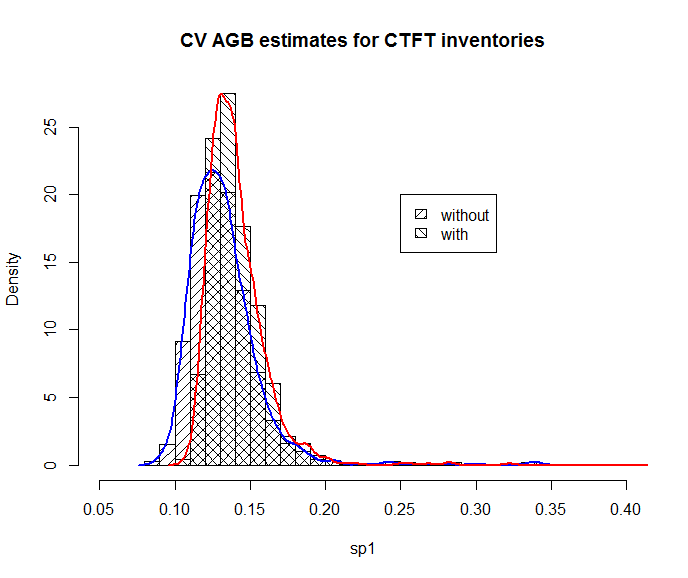


**Figures S3-1: Comparison of the uncertainties using 1000 simulations per plots, taking into account or without taking into account the allometry error.**


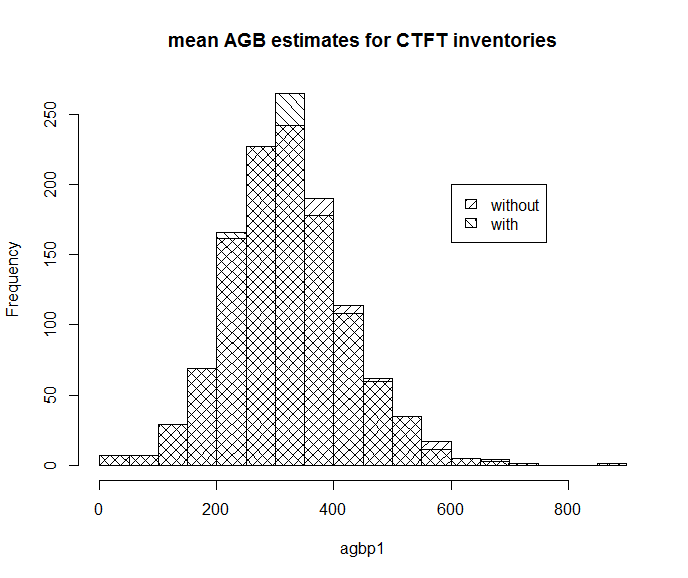

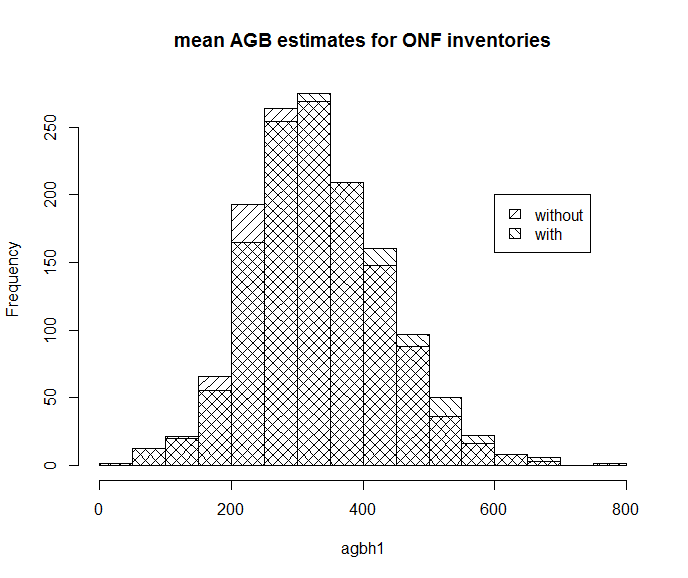


**Figures S3-2: Comparison of the mean of estimates using 1000 simulations per plots, taking into account or without taking into account the allometry error.**


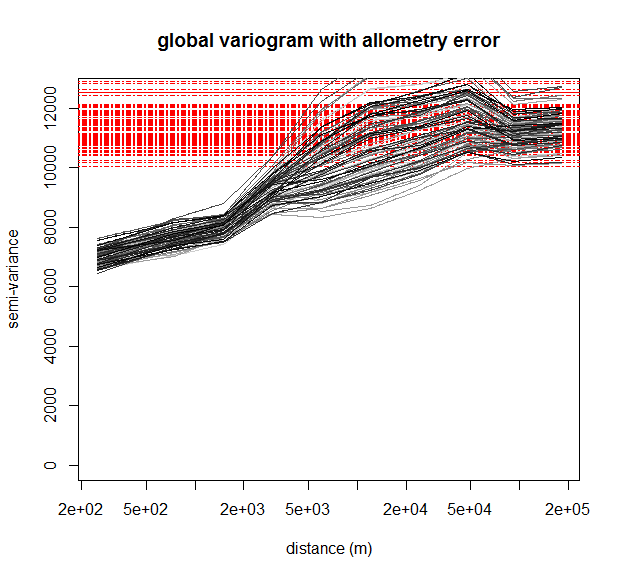

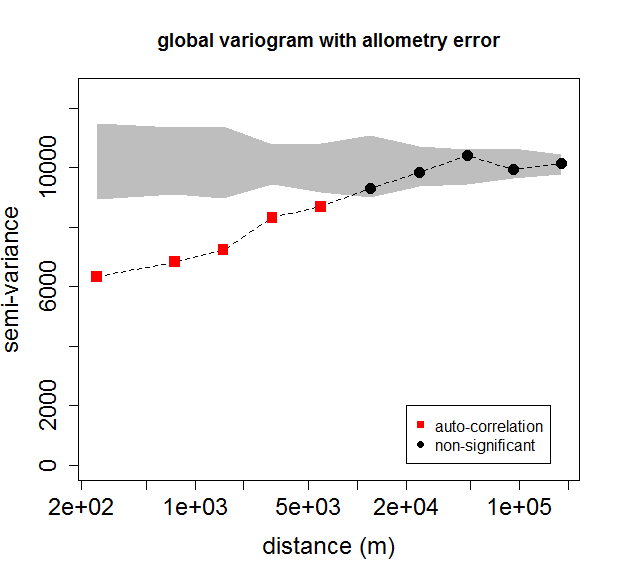


**Figures S3-3: Variograms for AGB estimates including allometry errors – the first graph compare variograms obtained from the 100 first simulations (red lines indicated the mean variance for each simulation dataset) – the second one is obtained from the mean AGB estimates including allometry errors.**

Integrating the error in the analysis of confidence intervals (IC_95%_) of local means reinforced our interpretation of the results. In fact, when modeling IC_95%_ in function of resolution and number of repetitions, the effect of resolution appeared to be not-significant (Figure S3-4). However, IC_95%_ appeared to be a little more important in this new version (e.g. 13% if 12 plots are used to estimate the local means at all resolution versus 12% in the precedent version for 2km-cells).


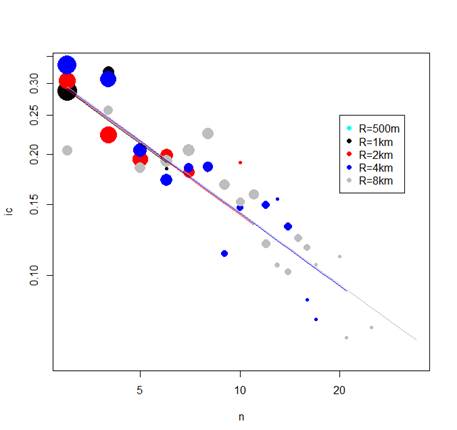


**Figure S3-4: Confidence interval at 95% of the local AGB means in function of resolution (R) and number of plots used to compute the local mean (n) – the circle represent the mean observed values (size is proportional to the number of observations) and lines indicate the fitted values.**

Last of all, we also propagated the uncertainties in our GLM analysis. For each simulation, we tested the complete linear model using all the environmental factors (+ the type of inventory i.e. ONF vs CTFT) and we analysed the distribution of the 1000 p-values obtained for each of them (Figure R1-5). Whatever the dataset used to model AGB variations, the effects of geomorphological landscapes (lands), and HAND were always very significant (max p-value 3.10^-7^ for lands and 7.10^-2^ for HAND). The effect of geology (geol) and vegetation class (veget) were significant in most of the cases (median p-value 0.06 for geol and 0.04 for veget). All other factors (including inventory type) appeared to be generally not-significant.

We then used these four selected factors to develop a linear model of prediction using only one half of our dataset for calibration and the other half for testing (similarly to our first version).We obtained nearly the same performance than in our first analysis : R²=0.09 as previously; RMSEP=97tC instead of 99tC. As previously, the residual error of the model appeared to be normally distributed (KS-test D=0.0312 p=0.17), with a limited heteroscedasticity (Beush-Pagan test BP=36.9, df=22, p=0.024). RMSE between the fitted values of this model and the fitted values of the previous model in our first analysis was 25Mg. The differences between the predictions of the two models were less than 6% in average (<8% for ¾ of the estimates with a maximum of 32%).

Residuals still showed a significant auto-correlation (Durbin-Watson test DW=1.74 p<0.001). The kriging analysis showed that the auto-correlation of the errors of prediction including allometry errors was shorter than in our first analysis (2.5km versus 7km) and was better predicted by a spherical function than by an exponential one. However, modelling the spatial error of prediction in a KR approach led to the same performance than in our first analysis (RMSEP equal to 90t.ha^-1^ as in the first analysis).


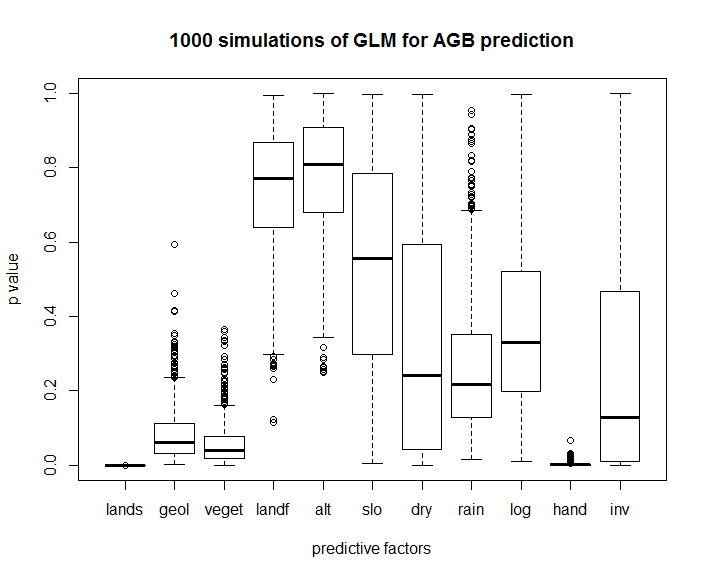


**Figure S3-5: Distribution of p-values obtained for each factors from the 1000 complete LM models based on the 1000 simulations.**

Finally, adding and propagating the uncertainty of local estimates in the analysis led to the same conclusions than in the simple analysis:

- Uncertainty at the plot scale is acceptable (less than 15%) even if forest inventory measurements are not very precise ;
- AGB spatial structure shows a high local variability and a weak auto-correlation limited to a few kilometers (<10km) ;
- Environmental effects account for a minor but significant part of AGB spatial variation with robust effects of geomorphological landscapes and Height Above the Nearest Drainage ;
- Enlarging the spatial resolution to compute local AGB estimates improves the confidence interval of local means ;
- Consequently, the accuracy of AGB maps is very limited at fine resolution because of the high variability and large uncertainty of local estimates but aggregating the prediction through larger cells improves the accuracy of the prediction maps.
- Consequently, the accuracy of AGB maps is very limited at fine resolution because of the high variability and large uncertainty of local estimates at local scale but aggregating the prediction at larger resolution improves the accuracy.
